# Meiotic Recombination in *Neurospora crassa* Proceeds by Two Pathways with Extensive Holliday Junction Migration

**DOI:** 10.1371/journal.pone.0147815

**Published:** 2016-01-26

**Authors:** Patricia Jane Yeadon, Frederick James Bowring, David E. A. Catcheside

**Affiliations:** School of Biological Sciences, Flinders University, Adelaide, South Australia, Australia; University of California, UNITED STATES

## Abstract

Analysis of thousands of *Δmsh*-2 octads using our fluorescent recombination system indicates that, as in other filamentous fungi, symmetric heteroduplex is common in the *his-3* region of *Neurospora crassa*. Symmetric heteroduplex arises from Holliday junction migration, and we suggest this mechanism explains the high frequency of His^+^ spores in heteroallelic crosses in which recombination is initiated *cis* to the *his-3* allele further from the initiator, *cog*^*+*^. In contrast, when recombination is initiated *cis* to the *his-3* allele closer to *cog*^*+*^, His^+^ spores are mainly a result of synthesis-dependent strand annealing, yielding asymmetric heteroduplex. Loss of Msh-2 function increases measures of allelic recombination in both *his-3* and the fluorescent marker gene, indicating that mismatches in asymmetric heteroduplex, as in *Saccharomyces cerevisiae*, tend to be repaired in the direction of restoration. Furthermore, the presence of substantial numbers of conversion octads in crosses lacking Msh-2 function suggests that the disjunction pathway described in *S*. *cerevisiae* is also active in Neurospora, adding to evidence for a universal model for meiotic recombination.

## Introduction

Meiotic recombination is a feature common to all sexually reproducing organisms, generating crossovers (COs), or reciprocal exchanges between chromosomes, which are required for chromosomes to segregate correctly during meiosis [1]. Recombination also ensures that variations within parental sequences come together in novel ways after meiosis, producing new material for natural selection. Rearrangement of sequence variations occurs not only as a result of new combinations of parental chromosomes and of crossing over between variations within each chromosome, but also from gene conversion [2, 3], where the copy number of one sequence increases at the expense of another.

A long-standing model of recombination was developed to explain what was thought to be a single pathway in *Saccharomyces cerevisiae*, in which a programmed double-strand break (DSB) is processed to yield a double Holliday junction that can be resolved in two ways to result in a CO or a non-crossover (NCO) [4, 5]. Current models, however, are derived from bodies of tetrad data from several organisms, including seminal studies using *Neurospora crassa* [3], *Ascobolus immersus* [6], *Sordaria fimicola* [7] and *S*. *cerevisiae* [8]. Reconciliation of apparently conflicting data obtained in *S*. *cerevisiae* has resulted in the conclusion that there are at least two pathways for crossing over during meiosis [9–13]. The “pairing” and “disjunction” pathways [13–15], known as the Class 2 and Class 1 CO pathways respectively [10, 16] also appear to apply to data from Sordaria [7], Drosophila [17–19] and Arabidopsis [20, 21]. It is thought that the Class 2 pathway is a direct descendant of the pathway for mitotic recombination, while the Class 1 pathway is a meiosis-specific modification evolved to regulate COs and ensure chromosome segregation [16].

The Class 1 pathway is dependent on the synaptonemal complex, requires Msh4/Msh5 proteins and generates interfering COs [9]. In *S*. *cerevisiae*, intermediate Class 1 molecules are short, contain fully ligated double Holliday junctions, and are believed to be resolved to yield only COs [10, 13, 15, 22]. The Class 2 pathway, in contrast, is Msh4/Msh5-independent with longer intermediates that yield predominantly NCOs as a result of synthesis-dependent strand annealing (SDSA) [23]. Invasion of the homolog by the 3′-overhanging end of a DSB [5] is followed by DNA synthesis to yield a transient intermediate that can be resolved by unwinding, as mediated by anti-crossover helicases such as Sgs1 [16]. Such unwinding results in newly synthesized DNA annealing to the initiating chromosome. It is possible for the 3′ end to invade the homolog more than once, leading to alternate patches of hetero- and homoduplex, a mechanism described as template-switching [23–26]. Any Holliday junction intermediates arising from the Class 2 pathway are resolved by protein complexes such as Mus81–Mms4 [27], yielding equal frequencies of NCOs and non-interfering COs [13, 15, 28].

Heteroduplex (hDNA) generated during recombination can be symmetric, present on both homologs adjacent to a Holliday junction, or asymmetric. Asymmetric hDNA is formed when a free 3′ end, resulting from digestion of the 5′ ends on either side of the DSB, invades the homologous chromosome, or when DNA, newly synthesized using the homolog as template, anneals with the initiating chromosome. Symmetric hDNA forms when a Holliday junction migrates further than the region in which DNA synthesis fills the 5′ gap, as the paired strands in each chromatid involved in recombination proceed to unwind and anneal with the homolog.

Evidence of the extent and nature of hDNA formed during recombination is often destroyed by the correction of mismatched DNA bases by mismatch repair (MMR) proteins. In eukaryotes, homologs of *Escherichia coli* MutS (Msh2, 3 and 6 proteins in *S*. *cerevisiae*) act as heterodimers to recognize mismatches, and the resultant complex then recruits a heterodimer of MutL homologs (*S*. *cerevisiae* Mlh1-3, Pms1) to repair the mismatch (reviewed in [23, 29, 30]). Thus, a useful strategy for a study of recombination is to disable MMR, leaving hDNA largely uncorrected. Since Msh2 is thought to be involved in recognition of all types of mismatch [29] yet results in little disturbance to meiosis, *MSH2* inactivation has been the usual choice [31]. However, it has been suggested Msh2 is required only in the Class 2 pathway [15], so we must therefore consider the possibility that only hDNA generated by the Class 2 pathway will lack correction in the absence of *MSH2*.

Using *S*. *cerevisiae* hybrids with genomes that differed at 46,000 or 52,000 positions, analysis of tetrads by 454 sequencing and high density microarrays indicated that about 90 COs and 45 NCOs occur in each meiosis [32, 33]. Even in an SK1/S288C hybrid where 62,000 SNPs distinguish the genomes, an average of 73 COs and 27 NCOs per meiosis was detected [31]. Since, despite the level of heterology, the combined NCO + CO frequency is similar to the estimated DSB frequency in a homologous diploid [34], the SK1/S288C hybrid was used to compare wild-type and *MSH2*-deletion meioses [31]. Lack of Msh2 was found to increase NCOs by a factor of three and to increase COs from 73 to 92 per meiosis. hDNA patterns suggested that not all NCOs occur by simple SDSA but that a substantial proportion (35%) are a result of Holliday junction dissolution by the combined action of a helicase and a type I topoisomerase [31]. Aberrant 4:4 segregation (Ab 4:4), indicating symmetric hDNA, was rarely seen despite the large number of markers and the absence of MMR [31]. Similarly, in an earlier study using poorly repaired mismatches at HIS4 [35], Ab 4:4 tetrads made up only 4.6% of non-Mendelian segregation (NMS, defined as any deviation from 4:4 segregation).

These data are in stark contrast to tetrad analyses using spore color mutations in *S*. *fimicola* in which the frequency of Ab 4:4 segregation is similar to that of gene conversion events, showing 6:2 segregation ([36, 37]; please note that henceforth we describe aberrant segregations as 5:3 or 6:2 regardless of which allele is present in excess). In both *S*. *fimicola* and *A*. *immersus* [38], Ab 4:4 segregation makes up 20–30% of NMS. However, in our recent study of 52,000 Neurospora asci, where alleles of a histone H1-GFP fusion gene substituted for spore color [39], we ignored Ab 4:4 asci, so the frequency of symmetric hDNA is currently unknown in Neurospora.

This GFP-based recombination reporter system [39, 40] has made it feasible to analyse recombination outcomes in thousands of asci relatively rapidly. Normal Mendelian segregation includes asci with GFP alleles in separate halves of the ascus or in pairs on either side, indicating that segregation of GFP has occurred at the first or second meiotic division respectively [39]. The latter is evidence that a CO has separated the centromere from the parental GFP allele before the first division of meiosis and so can be used as a measure of CO frequency between the centromere and the site of GFP integration on that chromosome. More rarely, an ascus will display NMS such as gene conversion or post-meiotic segregation (PMS), a result of hDNA formed during recombination, with or without mismatch repair respectively. In a *Δmsh-2* homozygote, hDNA generated in the Class 2 (pairing) pathway is expected to remain unrepaired. If conversion-type and restoration-type repair are equally likely, the frequency of 5:3 Class 2 pathway asci in a cross lacking Msh-2 function should be twice the frequency of 6:2 Class 2 asci when Msh-2 is active. If repair in the Class 1 (disjunction) pathway is *msh-2*-independent in Neurospora as it is in yeast [15], the frequency of 6:2 Class 1 asci should be unchanged by lack of Msh-2 function.

We have deleted *msh-2* by the split-marker method [41] in several different Neurospora strains, allowing isogenic analysis of the effect of Msh-2 on allelic recombination in *his-3*. This region was chosen because of the well-known recombination hotspot, *cog*, that initiates recombination about 3 kb from the 3′ end of the *his-3* coding sequence [42, 43]. There are two codominant alleles of the hotspot, *cog* and *cog*^*+*^, and recombination stimulated by either *cog* allele is suppressed by the unlinked *rec-2*^*+*^ gene [44]. In the absence of *cog*^*+*^, relief of *rec-2*^*+*^–mediated suppression increases allelic recombination only 4-fold, with no detectable effect on local crossing over. In contrast, a single copy of *cog*^*+*^ results in >40 times the level of allelic recombination and 6-fold more crossing over than seen in the *rec-2*^*+*^ cross, while two copies of *cog*^*+*^ increase allelic recombination >100-fold and crossing over 12-fold compared to the suppressed level [45]. It is therefore clear that recombination is rarely initiated at *cog* when the *cog*^*+*^ allele is present.

We have analyzed *Δmsh-2* octads with markers segregating on three Neurospora chromosomes for comparison with a previous study of wild-type octads [46] and compared allelic recombination frequencies in *Δmsh-2* and otherwise isogenic Msh2^+^ crosses. When combined with our GFP-based octad analyses, our data are supportive of a generally universal mechanism for recombination but suggests there are features yet to be fully described.

## Results

### The meiotic MMR function of Neurospora *msh-2*

**Fig 1:**
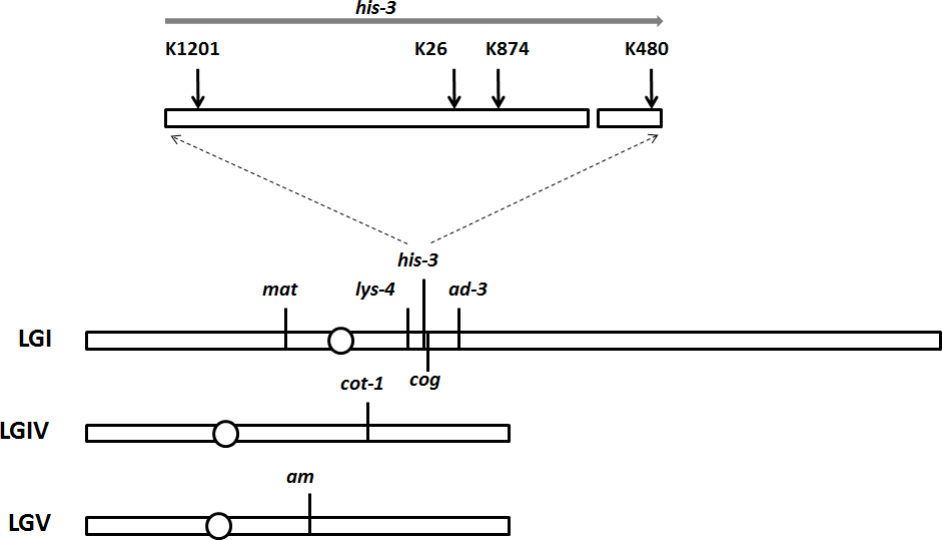
Genetic markers used in this study. The horizontal bars represent Neurospora linkage groups (LG) I, IV and V and the position of each centromere is indicated by a circle. The positions of the loci used as markers in this study are indicated above each linkage group, and the mutant sites within *his-3* are marked above the expansion indicating the coding sequence (top of figure). The small gap in the coding sequence indicates the intron. The exact location of the TM429 translocation [47] is unknown, but it is very close to the position of *his-3*^K874^ [48]. Note that the recombination hotspot *cog* is just to the right of *his-3* and the insertion site of the histone H1-GFP fusion gene [39] is between *cog* and *his-3*.

For comparison with 148 octads collected from previous analysis of an Msh-2^+^ cross (T12105 × T12282; Table 1 [46]), we collected 41 octads from an otherwise isogenic *msh-2* deletion cross (T12344 × T12342; Table 1). The crosses used for this analysis were heterozygous at four loci on LGI (*mat*, *lys-4*, *his-3* and *ad-3*; Fig 1) and at a single locus on each of LGIV and LGV (*cot-1* and *am* respectively; Fig 1). Of the 148 octads where MMR was wild-type, there were four showing NMS (6+:2M at *lys-4*, 6+:2M at *his-3*, 6+:2M and 2+:6M at *cot-1* [46]). In contrast, of the 41 octads from the cross lacking Msh-2 function, five displayed NMS, a greater frequency than in the Msh-2^+^ cross (p = 0.03). All the detected NMS octads from the *Δmsh-2* cross showed post-meiotic segregation (PMS; 5+:3M at *his-3*, 5+:3M and 3+:5M at *cot-1* and 5+:3M and 3+:5M at *am*), confirming that Neurospora MSH-2 has the meiotic MMR function predicted by similarity to the Msh2 protein of *S*. *cerevisiae*. Note that because the number of octads analyzed is small, the detected NMS events may not represent all such events in this cross.

**Table 1:** Neurospora strains. All *N*. *crassa* strains used in this study are listed below. Strain numbers are those used by the Catcheside laboratory, as many of these cultures are not held by the Fungal Genetics Stock Center.

Stock no.	Genotype
T10998	*A*, *his-3*^K874^, *cog*^*+*^, *ad-3; cot-1; rec-2*
T11089	*A*, *his-3*^K874^, *cog*, *ad-3; cot-1; am*, *rec-2*
T11802	*a*, *lys-4*, *his-3*^K1201^, *cog*; *cot-1; am*, *rec-2*
T11805	*a*, *lys-4*, *his-3*^K1201^, *cog*^*+*^; *cot-1; rec-2*
T12298	*A*, *his-3*^K874^, *cog*^*+*^, *ad-3; cot-1; rec-2; Δmsh-2*
T12299	*a*, *lys-4*, *his-3*^K1201^, *cog*^*+*^; *cot-1; rec-2; Δmsh-2*
T12105	*a*, *cog*^*+*^; *cot-1; rec-2*
T12282	*A*, *lys-4*, *his-3*^K480^, *cog*, *ad-3*; *am*, *rec-2*
T12342	*A*, *lys-4*, *his-3*^K480^, *cog*, *ad-3*; *cot-1; am*, *rec-2; Δmsh-2*
T12344	*a*, *cog*^*+*^; *cot-1; rec-2; Δmsh-2*
T12498	*A*, *his-3*^*+*^::*pccg-1*::*hH1*::*5′GFP*, *cog; rec-2*
T12515	*a*, *his-3*^*+*^::*pccg-1*::*hH1*::*3′GFP*, *cog; am*, *rec-2*
T12520	*rid*^rip4^, *a*, *his-3*^*+*^::*pccg-1*::*hH1*::*5′GFP*, *cog*^*+*^*; rec-2*
T12529T12571	*rid*^rip1^, *A*, *his-3*^*+*^::*pccg-1*::*hH1*::*GFP*^*+*^, *cog; am*, *rec-2rid*^rip4^, *a*, *his-3*^*+*^::*pccg-1*::*hH1*::*5′GFP*, *cog*^*+*^*; rec-2; Δmsh-2*
T12582	*rid*^rip1^, *A*, *his-3*^*+*^::*pccg-1*::*hH1*:: 3′GFP, *cog; am*, *rec-2*
T12651	*rid*^rip1^, *A*, *his-3*^*+*^::*pccg-1*::*hH1*:: 3′GFP, *cog; am*, *rec-2; Δmsh-2*
T12705/06/07	*A*, *his-3*^K874^, *cog*, *ad-3; cot-1; am*, *rec-2; Δmsh-2*
T12708/09/10	*a*, *lys-4*, *his-3*^K1201^, *cog*; *cot-1; am*, *rec-2; Δmsh-2*
T12711	*rid*^rip4^, *a*, *his-3*^*+*^::*pccg-1*::*hH1*::*5′GFP*, *cog*^*+*^*; rec-2; Δmsh-2*
T12713	*rid*^rip1^, *A*, *his-3*^*+*^::*pccg-1*::*hH1*::*GFP*^*+*^, *cog; am*, *rec-2; Δmsh-2*

The *am* allele is K314, *lys-4* is STL4, *cot-1* (colonial temperature-sensitive mutation) is C102t, and *ad-3* is K118.

### Meiotic silencing has no detectable effect on *msh-2* phenotype

A null *msh-2* mutant generated by repeat-induced point mutation [49] has been found to be recessive with respect to recombination [50]. A deletion mutant could however be dominant due to meiotic silencing, whereby a DNA coding sequence that lacks a partner on the homologous chromosome is prevented from being expressed during meiosis [51, 52].

**Fig 2:**
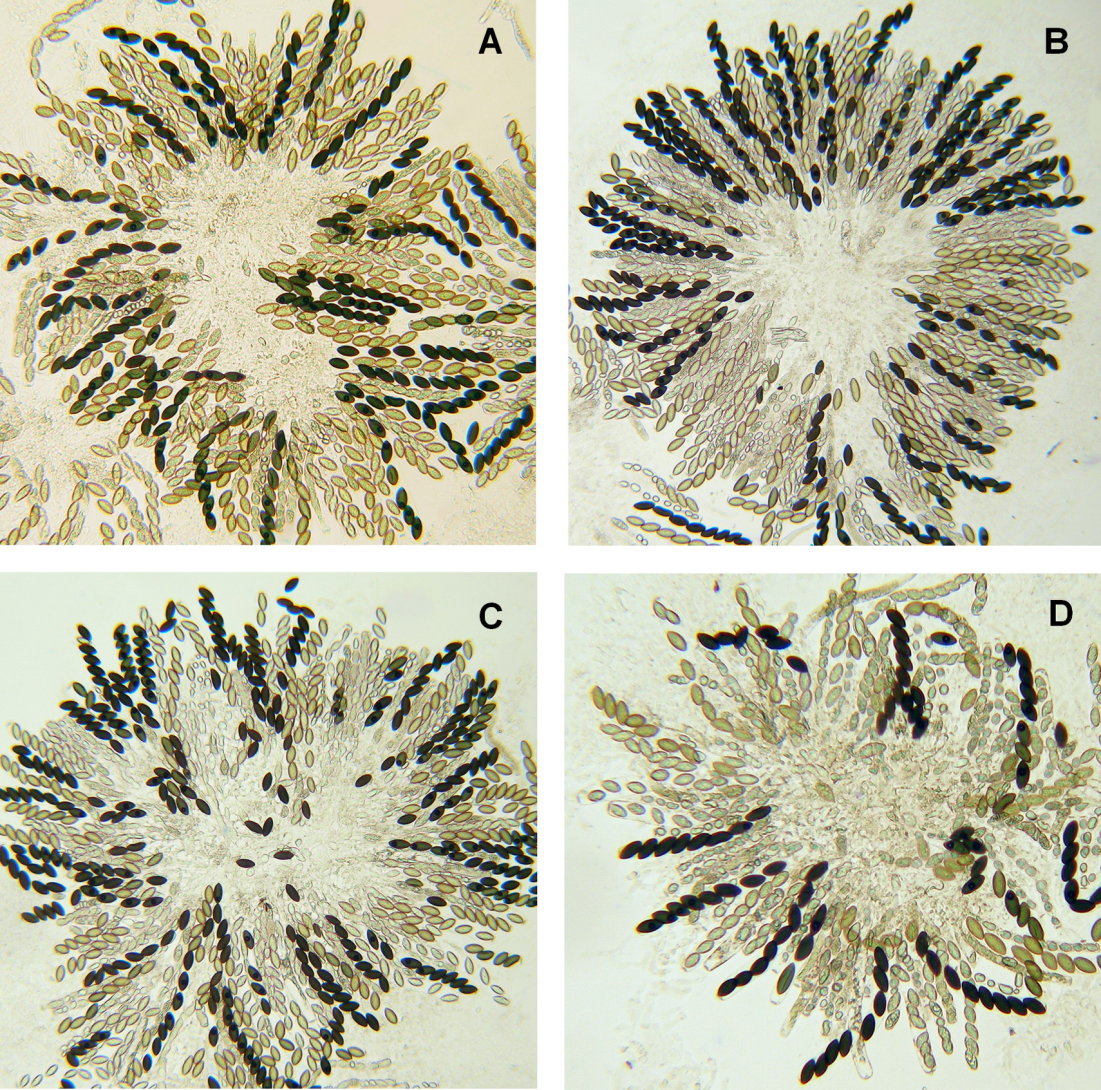
The effect of *msh-2* deletion on meiosis is recessive. Whether the *msh-2* parent is the male (B) or the female (C), rosettes from heterozygous crosses appear the same as the homozygous wild-type (A). In contrast, homozygous deletion (D) results in abnormal spores and a reduction in fertility, although there are some asci with eight viable spores.

Meiosis is subtly disturbed in crosses homozygous for *Δmsh-2*, resulting in deformed and infertile asci (Fig 2D) relative to the wild-type cross (Fig 2A), and a slight delay in sporogenesis. In addition, the frequency of His^*+*^ progeny of a cross heteroallelic for *his-3*^K874^ and *his-3*^K1201^ mutations [48] is significantly increased in crosses homozygous for *Δmsh-2* relative to otherwise isogenic crosses homozygous for *msh-2*^*+*^, while crosses heterozygous *Δmsh-2*/*msh-2*^*+*^ (Table 2) give His^*+*^ spore frequencies indistinguishable from those of the *msh-2*^*+*^ homozygote. Regardless of which parent is deleted for *msh-2*, perithecia from *Δmsh-2*/*msh-2*^*+*^ heterozygotes appear normal (Fig 2B and 2C). Thus, there is no detectable silencing of *msh-2*^*+*^ in either hemizygote, and *Δmsh-2* behaves as classically expected for a recessive mutant.

**Table 2:** Loss of Msh-2 function increases allelic recombination in *his-3* only when *cog*^*+*^ is *cis* to the *his-3* allele closer to it. The His^+^ frequency is increased by a factor of 1.5 in *his-3*^*K874*^ *cog*^*+*^ × *his-3*^*K1201*^ *cog*^*+*^ crosses (Fig 3A), while remaining unchanged by heterozygosity for *Δmsh-2*. However, although lack of Msh-2 function similarly increases His^+^ frequency in *his-3*^*K874*^ *cog*^*+*^ × *his-3*^*K1201*^ *cog* crosses (Fig 3B), there is no effect on His^+^ frequency in *his-3*^*K874*^ *cog* × *his-3*^*K1201*^ *cog*^*+*^ crosses (Fig 3C).

Cross	Genotype	His^+^	p value
***his-3***^***K874***^ ***cog***^***+***^ ***× his-3***^***K1201***^ ***cog***^***+***^	*Msh-2*^*+*^	946	
	*Msh-2*^*+*^*/Δmsh-2*	975	0.370
	*Δmsh-2*	1460	0.001
***his-3***^***K874***^ ***cog***^***+***^ ***× his-3***^***K1201***^ ***cog***	*Msh-2*^*+*^	690	
	*Δmsh-2*	1228	0.020
***his-3***^***K874***^ ***cog × his-3***^***K1201***^ ***cog***^***+***^	*Msh-2*^*+*^	390	
	*Δmsh-2*	427	0.630

All crosses are between strains carrying *his-3*^*K874*^ and *his-3*^*K1201*^ alleles and are homozygous for *rec-2*. **His**^**+**^ is the mean frequency of histidine-independent progeny per 10^5^ viable spores. Strains used to obtain these data are T10998, T11089, T11801, T11805, T12298, T12299 and T12705-T12710 (Table 1; S1 Table).

### Loss of Msh-2 function increases allelic recombination–under most circumstances

**Fig 3:**
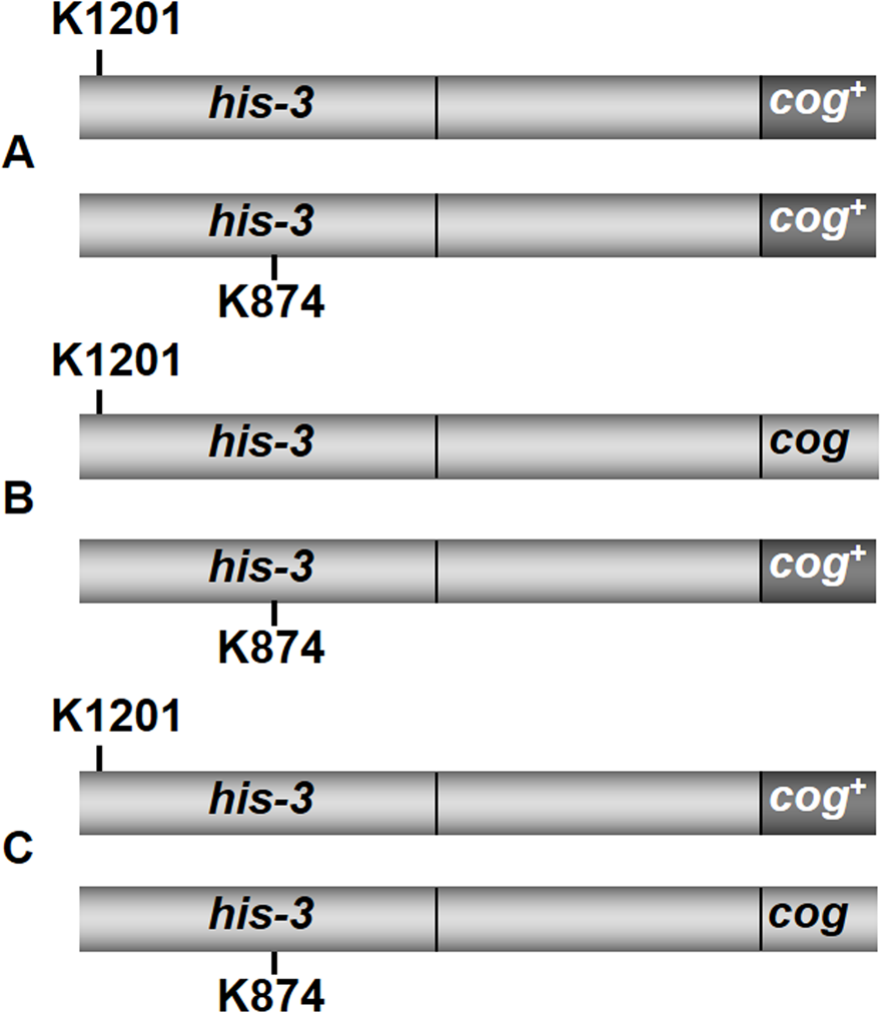
Possible arrangements of *cog*^*+*^, *cog* and alleles *his-3*^K1201^ and *his-3*^K874^. The centromere is to the left of the figure and *ad-3* is to the right. The figure is not to scale. In A, the cross is homozygous for *cog*^*+*^; in B, *cog*^*+*^ is *cis* to *his-3*^K874^, the mutant site closer to *cog*; while in C, *cog*^*+*^ is *cis* to *his-3*^K1201^, the mutant site further from *cog*.

When both chromosomes carry *cog*^*+*^ in a cross that is heteroallelic for mutations in *his-3* (Fig 3A), the His^*+*^ spore frequency is increased ~1.5-fold by loss of Msh-2 function (Table 2). In crosses heterozygous for *cog*^*+*^, when the *his-3* allele closer to *cog* is *cis* to *cog*^*+*^ (Fig 3B), the frequency of His^*+*^ spores is similarly increased by loss of Msh-2 function (Table 2). In contrast, when the *his-3* allele closer to *cog* is *trans* to the only copy of *cog*^*+*^ (Fig 3C), the frequency of His^*+*^ spores is unchanged by loss of Msh-2 function (Table 2).

**Fig 4:**
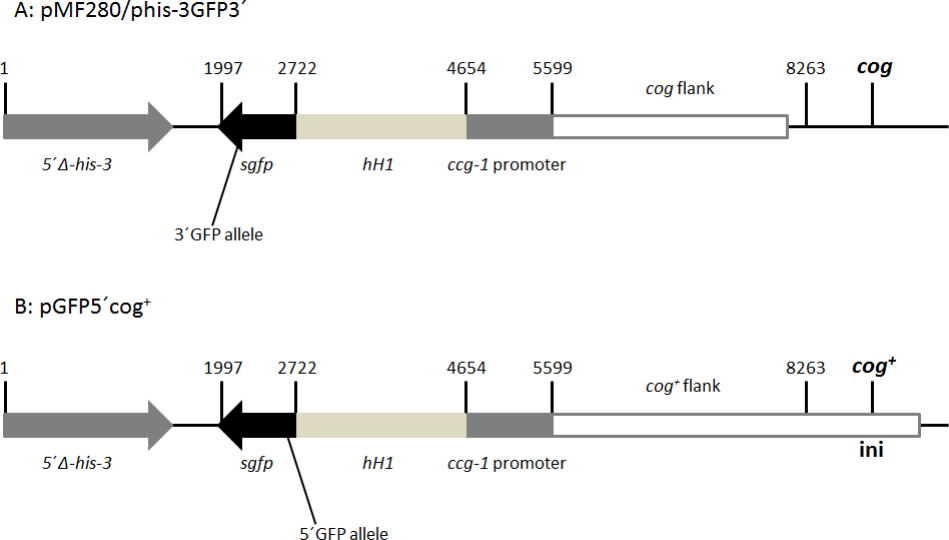
GFP constructs inserted at *his-3*. Plasmids are based on pMF280 [38], shown at top of figure, in which the arrows indicate the directions of transcription for the *his-3* and *sgfp* coding sequences. A mutation (substitution of T for A at nucleotide 628) was placed in the 3′ end of the *sgfp* sequence in pMF280 to give phis-3GFP3′ [38]. pGFP5′cog^+^ (lower part of figure) was made by joining the left side of pMF280, including the 5′-truncated *his-3* sequence, *sgfp*, hH1 and the *ccg-1* promoter, to sequences amplified from a *cog*^*+*^ strain [39] and a mutation (substitution of T for G at nucleotide 26) placed in the 5′ end of the *sgfp* sequence. The constructs were targeted to *his-3* by transformation of *his-3* mutant strains and selection for growth without histidine. “**ini**” indicates the putative recombination initiation site within *cog*^*+*^ [24], so recombination is initiated about 6 kb from the GFP5′ mutation.

In a cross in which one strain has a 5′GFP and the other a 3′GFP non-fluorescent mutant construct, each inserted between *his-3* and *cog* (Fig 4), the frequency of fluorescent spores is a measure of allelic recombination within the GFP construct. In these crosses, *cog*^*+*^ is *cis* to the marker closer to it (5′GFP). Although the *Δmsh-2* and *msh-2*^*+*^ homozygotes are not isogenic as the *Δmsh-2* GFP strains were extracted from crosses, the fluorescent spore (GFP^+^) frequency is increased by loss of Msh-2 function to a degree (1.4-fold; Table 3) similar to that of the His^*+*^ spore frequency in heteroallelic *his-3* crosses (Table 2). Thus, the increase in allelic recombination at *his-3* in the absence of Msh-2 function is not peculiar to *his-3*.

**Table 3:** Allelic recombination in GFP is increased by loss of Msh-2 function. In each cross (*msh-2*^*+*^ is T12498 × T12515 and *Δmsh-2* is T12571 × T12651; Table 1), one chromosome carries a 3′GFP and the other a 5′GFP construct, each inserted at the same position between *cog* and *his-3*. PMS is increased 13-fold (p < 0.0001) and frequency of GFP^+^ spores is increased 1.4-fold (p = 0.003) by loss of Msh-2 function.

Octad type	*msh-2*^*+*^ asci	Asci %	*Δmsh-2* asci	Asci %
No fluorescence	13444	99.66	18158	99.15
**1+:7M**	8	0.06	138	0.75
**2+:6M**	34	0.25	15	0.08
**3+:5M**	0	0	4	0.02
**4+:4M**	4	0.03	0	0
**Ab 2+:6M**	0	0	1	0.005
Total	13490	100	18316	100
**C ± SE**	0.37 ± .052		0.89 ± .069	
**GFP**^**+**^ **± SE**	0.68 ± .025		0.97 ± .026	

**C ± SE** is the calculated frequency of conversion events in each 100 asci ± the standard error of that frequency. **GFP**^**+**^ **± SE** is the calculated frequency of GFP^+^ spores in each 100 spores ± the standard error of that frequency.

### NMS of GFP^+^ appears increased by loss of Msh-2 function

In a cross in which one strain has a 5′GFP (M) and the other a GFP^+^ (+) construct, each inserted between *his-3* and *cog*, the pattern of fluorescence in an ascus indicates if a recombination event has occurred in the meiosis from which the ascus was derived [39]. We have data (Table 4) from crosses that differ only in that wild-type *msh-2* in T12529 and T12520 has been replaced by *hph*, conferring hygromycin resistance and generating the otherwise isogenic *Δmsh-2* strains T12713 and T12711 respectively (Table 1). These crosses are, as in the 5′GFP × 3′GFP crosses above, heterozygous *cog/cog*^*+*^, with *cog*^*+*^ closer and *cis* to the 5′GFP mutation.

**Table 4:** GFP analysis of *Δmsh-2* homozygotes reveals a range of recombination outcomes near *his-3*. In each cross (*msh-2*^*+*^ is T12529 × T12520 and *Δmsh-2* is T12713 × T12711; Table 1), one chromosome carries a GFP^*+*^ and the other a 5′GFP construct, each inserted at the same position between *cog* and *his-3*. PMS is increased ~20-fold (p < 0.0001) and second division segregation 1.5-fold (p < 0.0001) by loss of Msh-2 function, while conversion frequency is unchanged (p = 0.5).

Octad type	*msh-2*^*+*^ asci	Asci %	*Δmsh-2* asci	Asci %
**D1**	10384	87.54	5530	79.06
**D2**	1378	11.62	1261	18.03
**6+:2M**	60	0.50	30	0.43
**5+:3M**	5	0.04	82	1.17
**7+:1M**	0	0	4	0.06
**8+:0M**	3	0.03	1	0.01
**Ab 5+:3M**	0	0	2	0.03
**Ab 6+:2M**	2	0.02	2	0.03
**Ab 4+:4M**	30	0.25	83	1.18
Total	11862		6995	
**CX ± SE**	5.9 ± 0.19		9.3 ± 0.50	
**C ± SE**	0.59 ± 0.07		1.19 ± 0.13	

**D1** is the number of asci showing first division segregation of GFP and **D2** the number with second division segregation. **CX** represents the percentage of crossover events between the centromere and GFP while **C** indicates the frequency of conversion asci per 100 asci (± the standard errors of each frequency).

The frequency of 6+:2M asci is little altered by loss of Msh-2 function (Table 4), although the spectrum of NMS is very different from when Msh-2 is active. The frequency of unrepaired mismatches (5+:3M, 7+:1M, Ab6+:2M, Ab5+:3M) is much increased by loss of Msh-2 function, as is the frequency of unrepaired symmetric hDNA (Ab 4:4). Overall, loss of Msh-2 function increases NMS ~3.5-fold, most of which is equally divided between 5+:3M and Ab 4:4.

## Discussion

Our analyses have generated data that suggest a number of conclusions. Firstly, the frequency of octads showing PMS is increased 13-fold by loss of Msh-2 function (Table 4), so repair of a substantial proportion of hDNA is *msh-2*-dependent in Neurospora, as it is in yeast. Secondly, both the ~3-fold increase in the total frequency of NMS (Table 4) and the ~1.5-fold increase in recombinant His^*+*^ spores in the absence of Msh-2 function (Table 2) indicate MMR at *his-3* and GFP is strongly biased in the direction of restoration. The mismatches in *his-3* and GFP are all >3 kb from the putative initiation site *cog*^*+*^ and mismatches >1 kb from a DSB are also preferentially restored in yeast [53, 54]. Finally, the frequency of 6:2 segregation of the 5′GFP mutation is little changed by loss of Msh-2 function (Table 4), indicating the existence of a DSB repair pathway in which MMR is *msh-2*-independent, as postulated for yeast [15]. Together, these data are supportive of the two pathway model for meiotic recombination [9–13], adding to the evidence that it may be universal.

On the other hand, additional data make the picture more complex. In a *cog*/*cog*^*+*^ heterozygote, recombination is known to be overwhelmingly initiated at *cog*^*+*^ [24, 45, 47]. In crosses heterozygous *cog*/*cog*^*+*^ and heteroallelic for *his-3* point mutations, most His^*+*^ spores (^2^/_3_ to ^3^/_4_) result from conversion of the site *cis* to *cog*^*+*^, irrespective of whether that site is the one closer to or further from *cog*^*+*^ [45]. In the case of *his-3*^TM429^, a mutation generated by a reciprocal translocation between LGI and LGVII that separates the 5' and 3' ends of the *his-3* coding sequence, the *his-3*^TM429^ allele cannot experience gene conversion. In a *his-3*^TM429^ heterozygote in which the point mutant is further from *cog* than TM429, *cog*^*+*^-stimulation of recombination absolutely requires the point mutant to be *cis* to *cog*^*+*^ [47]. In this case, conversion on the far side of TM429 from *cog* is thought to occur by template-switching [23, 24], whereby recombination initiated on one chromosome proceeds via SDSA using as template not only the homolog but also the sister [24]. But if the point mutant is closer to *cog* than TM429, *cog*^*+*^-stimulation of recombination occurs even if the point mutant is not *in cis* to *cog*^*+*^ [24, 47].

We have already noted that in *his-3*^K1201^ × *his-3*^K874^ crosses, if the cross is homozygous for *cog*^*+*^ (Fig 3A), or if the only copy of *cog*^*+*^ is *cis* to *his-3*^K874^, the mutant site closer to the initiator (Fig 3B), loss of Msh-2 function increases recombinant His^*+*^ spores about 1.5-fold (Table 2). However, if the only copy of *cog*^*+*^ is *trans* to *his-3*^K874^ (Fig 3C), loss of Msh-2 function has no effect on recombination frequency (Table 2). In addition, the need for conversion of a site more distant from *cog*^*+*^ without co-conversion of the closer site only reduces the frequency of His^*+*^ spores to about 56% of the frequency observed with a single copy of *cog*^*+*^ *cis* to *his-3*^K874^ (Table 2).

It is not immediately obvious how conversion of the *his-3* allele more distant from *cog*^*+*^ occurs at such high frequency, since it is evident that recombination is initiated predominantly at *cog*^*+*^ [24, 45, 47] and the initiating chromosome has been shown to be the usual recipient of information [47, 55]. One mechanism that springs to mind is template-switching [23, 24], the same way that recombination crosses the TM429 translocation breakpoint [24]. However, in a cross between *his-3*^TM429^ and *his-3*^K1201^, the His^+^ frequency is ~0.01% [47], compared to ~1% in a cross between the similarly positioned *his-3*^K874^ [48] and *his-3*^K1201^ (Table 2; [45]). Since the requirement to cross the breakpoint leads to a 100-fold decrease in His^+^ spores, this suggests that template switching is infrequent and thus likely to make little contribution to the frequency of His^+^ spores when *cog*^*+*^ is *trans* to *his-3*^K874^.

Data from crosses heterozygous 5′GFP/GFP^+^ indicate the frequency of Ab 4:4 is high even in *msh-2*^*+*^ NMS octads in Neurospora (0.25% of octads and 30% of NMS; Table 4), in contrast to studies using *S*. *cerevisiae*, in which Ab 4:4 has rarely been detected [31]. Although some may be a result of spindle slippage during meiosis or of spores slipping past each other in an ascus [56], loss of Msh-2 function increases Ab 4:4 frequency to 1.2% of octads (46% of NMS). Thus symmetric hDNA appears to be very common at *his-3* in Neurospora, occurring at a similar level to that demonstrated in other filamentous fungi [36–38]. The frequency of symmetric hDNA suggests another mechanism that may explain the frequency of conversion of sites *trans* to *cog*^*+*^, that of Holliday junction migration (HJM).

**Fig 5:**
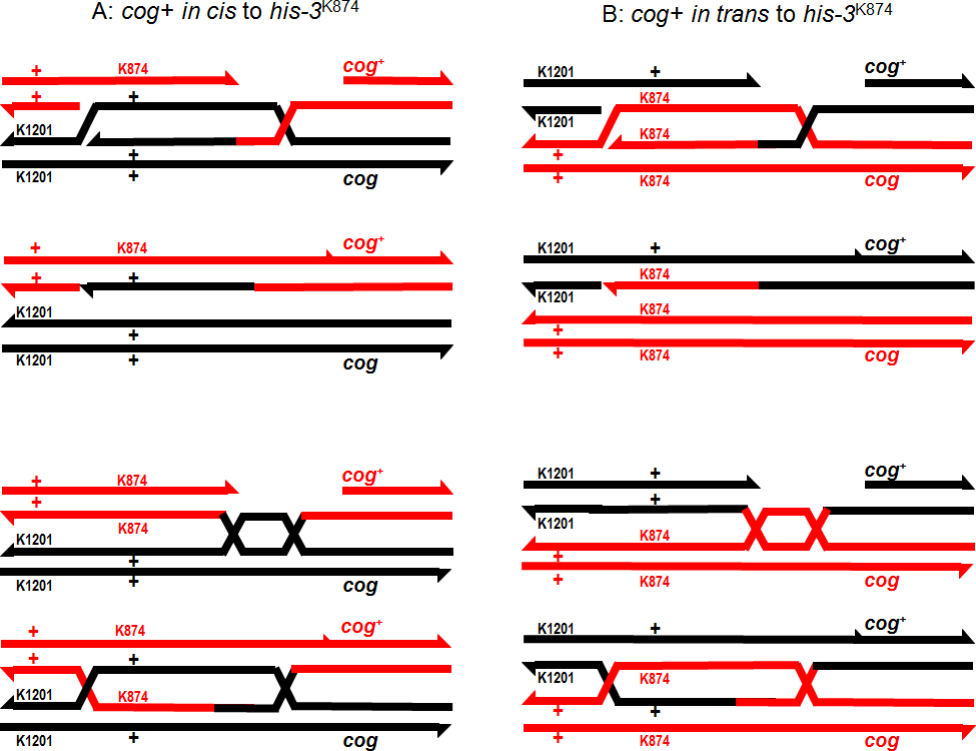
Mechanism of generation of His^*+*^ spores depends on the relative positions of *cog*^*+*^ and the *his-3* mutant alleles. The centromere is to the left of the figure and *ad-3* is to the right. Recombination of *his-3* alleles is initiated by a DSB within the *cog*^*+*^ region and the 5′ ends of the break are resected to give 3′ overhanging ends [57]. A 3′ end invades the homolog, displacing the strand of like polarity, and DNA synthesis proceeds to fill the gap [57].

The probability that a His^+^ spore will be produced by a meiotic event depends on how often recombination is initiated at *cog*^*+*^ [24, 45], the chance that hDNA includes the closer mutant site in *his-3* [57] or covers both mutant sites, and how mismatches within *his-3* are repaired. In crosses heterozygous for *cog*^*+*^, the sum of these probabilities depends on whether the mutant site closer to *cog* is on the same chromosome as *cog*^*+*^ or not (Fig 5). When *cog*^*+*^ is *cis* to *his-3*^K874^, SDSA can result in a His^+^ spore (Fig 5A) but not when *cog*^*+*^ is *trans* to *his-3*^K874^ (Fig 5B). Conversely, whether *cog*^*+*^ is *trans* or *cis* to the closer mutant site, HJM is equally likely to produce symmetric hDNA covering only the closer mutant site (*his-3*^K874^ in Fig 5), followed by resolution of the junction between the mutant sites to yield a His^+^ spore (Fig 5). Although symmetric hDNA covering both mutant sites can also result in a His^+^ spore if the two sites are repaired in opposite directions, this mechanism would result in a decrease in His^+^ spores in the absence of Msh-2 function.

At this point, synthesis may continue (top section of A and B), generating a migrating D-loop (see [57]). Ligation of ends does not occur at this early stage, so the recombination intermediate can unwind, giving conversion without a crossover (SDSA). Note that DNA synthesis can switch readily between the homolog and the initiating chromatid (or the sister, not shown in this figure), provided that the sequences of the homologs are sufficiently similar for binding of the end to occur [57]. If the SDSA structure (A top section) is unrepaired, the meiosis yields a single His^+^ spore. Mismatch repair of the *his-3*^K874^ mutation to wild-type can give two His^+^ spores, while repair of the newly synthesized strand in the hDNA will result in no His^+^ spore.

Alternatively, ligation of ends results in Holliday junctions, one or both of which may migrate towards *his-3* (A and B, lower sections). If the left-most junction is resolved between the sites of the *his-3*^K874^ and *his-3*^K1201^ mutations, this can yield a single His^+^ spore. Once again, mismatch repair can increase the number of His^+^ spores to two or reduce it to zero, depending on the direction of repair. Note that when *cog*^*+*^ is *cis* to *his-3*^K874^ (A), both SDSA and Holliday junction migration can result in His^+^ spores, while if *cog*^*+*^ is *trans* to *his-3*^K874^ (B), His^+^ spores cannot be generated by SDSA.

As described in the two pathway model for recombination in *S*. *cerevisiae* [13, 14], mismatches in hDNA from the pairing pathway are usually restored to 4:4 by a *MSH2*-dependent mechanism [15]. Thus if most of the asymmetric hDNA in Neurospora were a product of the pairing pathway, loss of Msh-2 function would lead to recovery of unrepaired hDNA and 5:3 segregation, which appears to be the case (Tables 3 & 4). The two pathway model [15] also suggests that the 6:2 segregation seen in *Δmsh-2* octads is a result of the disjunction pathway, in which MMR is *MSH2*-independent, and such resolutions would be predicted to be as COs [15]. Our data do not conflict with this hypothesis but suggest that symmetric hDNA is also part of the *msh-2*-dependent pairing pathway, since loss of Msh-2 function increases the frequency of Ab 4:4 and 5:3 segregation to similar extents (Table 4).

We suggest that SDSA may be responsible for the majority of His^+^ spores when *cog*^*+*^ is homozygous, or when *cog*^*+*^ is *cis* to the mutant site closer to it (Table 2). SDSA with template switching is known to be responsible for His^+^ spores in crosses heteroallelic for *his-3*^TM429^ when the point mutant is further from *cog*^*+*^ than the translocation [24]. Our data suggest that repair of mismatches in asymmetric hDNA shows about a 4-fold bias towards restoration. Perhaps the free end in the unligated intermediate [15] may direct repair to the newly synthesized strand.

We also suggest that junction migration is responsible for the high frequency of His^+^ spores from heteroallelic crosses when *cog*^*+*^ is *trans* to the closer mutant site. Since loss of Msh-2 function does not alter His^+^ spore frequency in this situation (Table 2, Fig 3C), it seems likely that the direction of repair in symmetric hDNA is unbiased, although repair direction may be determined by the direction of cutting of a Holliday junction when the intermediate is resolved, perhaps by Mus81–Mms4 [27].

In general, recombination in Neurospora appears to follow the “rules” of the two pathway model [13, 15], with evidence for both the pairing and the disjunction pathways and suggesting *MSH2*-independence of the latter pathway in Neurospora as in *S*. *cerevisiae*. Our data indicate that *MSH2*-dependent MMR of mismatches generated by recombination initiated at *cog*^*+*^ is biased in the direction of restoration to normal 4:4 segregation, as seen in yeast studies of markers distant from an initiating DSB site [58, 59]. However, this analysis has also revealed the substantial contribution of HJM to gene conversion at *his-3* of Neurospora, a completely different picture to that seen in *S*. *cerevisiae*, where HJM is rarely detected [31, 35]. Thus, a gradient in HJM may substitute for or add to conversion gradients resulting from changes in the direction of MMR [58], from the extent of hDNA formation during DNA repair synthesis [60] or from MMR-regulated hDNA rejection [59, 61]. Since HJM is likely to have a significant impact on recombination in other filamentous fungi and conceivably in higher eukaryotes, the existence of this feature in Neurospora indicates the need to study recombination in a wide range of model organisms.

## Methods

### Culture and media

Culture methods were as described previously [62], except that crosses were supplemented with 200μg/ml l-histidine, 500μg/ml l-alanine, 500μg/ml l-arginine, 200μg/ml adenine and 400μg/ml l-lysine as required. Vegetative cultures were supplemented with 200μg/ml l-histidine, 500μg/ml l-arginine, 500μg/ml l-alanine, 400μg/ml adenosine and 400μg/ml l-lysine as required. Recombination assays and crosses on solid media were as described previously [45, 46]. Microscopy and data collection were as described previously [39]. Transformation of Neurospora conidia was by electroporation [63].

### Generation of DNA constructs for deletion of *msh-2*

Left and right flanks were amplified from cosmid G4:D9 [64] using primer pairs msh2Lfwd (ATCAGTCTCCTCCATCATACCC) with msh2Lrev (gtcgtgactgggaaaaccctggcgAAGTCGGATTGTTAGGAAGTCG) and msh2Rfwd (tcctgtgtgaaattgttatccgctACTGAGTGGTGATGGTGGAC) with msh2Rrev (TTCCCTTTTCCCCTTTCC). Left and right flank constructs were respectively fused to incomplete overlapping portions (HY and YG) of the hygromycin phosphotransferase (*hph*) cassette, using fusion PCR [65]. Fusion of the left flank and HY used primer pairs msh2Lfwd with HY (NLC37: GGATGCCTCCGCTCGAAGTA; [41]), while the left flank fusion used primers YG (NLC38: CGTTGCAAGACCTGCCTGAA; [41]) with msh2Rrev.

### Construction of strains

Original strains with GFP inserted at *his-3* (T12515, T12520 and T12529; Table 1), contain the 3′GFP, 5′GFP and GFP^+^ constructs respectively (Fig 4), and were made as described previously [39]. The 5′ allele is a substitution of T for G at nucleotide 26 of the GFP coding sequence (p.Glu6*), while the 3′ allele is a substitution of T for A at nucleotide 628 (p.Lys210*) [39].

Most *msh-2* deletion strains were made using the standard split-marker process [41], by transformation with the left and right deletion constructs (described above), and selecting for growth on hygromycin. Strains transformed were T10998, T11805, T12105, T12282, T12520 and T12529, to give T12298, T12299, T12344, T12342, T12711 and T12713 respectively (Table 1). After separation of heterokaryons [66], homokaryotic transformants were confirmed by Southern analysis. Additional *msh-2* deletion strains were extracted as hygromycin-resistant progeny of crosses. T12705, T12706 and T12707 are from a cross between T11089 × T12299; T12708, T12709 and T12710 are from T11802 × T12298; T12571 from T12298 × T12520 and T12651 from T12299 × T12582 (Table 1).

### Statistical analysis of recombination data

For analysis of octad data using genetic markers, χ^2^ or Fisher’s exact tests were used to assess the probability that the distribution of genotypes in the progeny of the mismatch-repair competent and otherwise isogenic *msh-2* deletion crosses could differ by chance.

For data obtained from asci segregating GFP alleles, frequency of conversion (C) within GFP was calculated as conversion events per 100 asci, where an ascus that has experienced a single conversion event (6+:2M, for example) is considered as one, while if two events have happened in the same ascus (8+:0M), that ascus is counted as two. So for example, if the total number of asci is 1000, of which there were four 6+:2M asci and three 8+:0M asci, the frequency of conversion is [(4 + (2 x 3))/1000] x 100%, or 1%. Aberrant 4+:4M asci (+++M+MMM and ++MMM+M+, for example) were included in the total ascus count but were not considered to be conversion asci. As the 5′GFP mutant is *cis* to *cog*^*+*^, 2+:6M asci are relatively uncommon and because they cannot be differentiated from asci with dead spores, they are routinely not scored [39]. Standard errors (SEs) of proportions were calculated according to the following formula–SE = √[*p*(1-*p*)/*n*] where *p* is the proportion of recombinant asci (as a fraction of unity) and *n* the number of asci scored.

For recombination assay data, two-tailed t-tests were used to compare frequencies of His^+^ spores, with each frequency transformed (p → sin^-1^√p) prior to comparison [67]. To compare allelic recombination frequencies in crosses between mutant GFP alleles with those in crosses between mutant *his-3* alleles, the proportion of recombinants per 10^5^ spores was calculated as the number of individual fluorescent spores divided by the total number of spores, multiplied by 10^5^. SEs were calculated as described for asci segregating mutant and wild-type GFP alleles (above), except that *p* is the proportion of recombinant spores (as a fraction of unity) and *n* the number of spores scored.

## Supporting Information

S1 Table “A” is the number of colonies on medium lacking histidine (selective plates), while “C” is the number of colonies on fully supplemented medium (viable count). In all crosses except those marked thus *, the number of spores plated on A was 200 times that plated on C.
(DOCX)
