# Supplementary material for: Meiotic Recombination in Neurospora crassa Proceeds by Two Pathways with Extensive Holliday Junction Migration
Source: PLoS One. 2016 Jan 26;11(1):e0147815. doi: 10.1371/journal.pone.0147815 (PMC4727923; doi:10.1371/journal.pone.0147815)
Supplement: S1 Table — In all crosses except those marked thus *, the number of spores plated on A was 200 times that plated on C. (DOCX) [file pone.0147815.s001.docx]

**S1 Table.** “A” is the number of colonies on medium lacking histidine (selective plates), while “C” is the number of colonies on fully supplemented medium (viable count). In all crosses except those marked thus *, the number of spores plated on A was 200 times that plated on C.

| **Cross** | **A** | **C** | **His^+^/10^5^** | **SE** |  |
| --- | --- | --- | --- | --- | --- |
| T10998 x T11805 | 913 | 1087 | 840 | 38 | ***his-3^K874^ cog^+^ x his-3^K1201^ cog^+^*** |
| T10998 x T11805 | 480 | 480 | 1000 | 65 | *Msh-2^+^/Msh-2^+^* |
| T10998 x T11805 | 461 | 506 | 911 | 59 |  |
| Mean |  |  | 917 |  |  |
|  |  |  |  |  |  |
| T10998 x T12299 | 443 | 425 | 1042 | 71 | *Msh-2^+^/Δmsh-2* |
| T12298 x T11805 | 447 | 468 | 955 | 63 |  |
| T12298 x T11805 | 351 | 378 | 929 | 69 |  |
| Mean |  |  | 975 |  |  |
|  |  |  |  |  |  |
| T12298 x T12299 | 1212 | 841 | 1441 | 65 | *Δmsh-2/Δmsh-2* |
| T12298 x T12299 | 615 | 442 | 1391 | 87 |  |
| T12298 x T12299 | 628 | 406 | 1547 | 99 |  |
| Mean |  |  | 1460 |  |  |
|  |  |  |  |  |  |
| T10998 x T11801 | 433 | 120 | 541 | 56 | ***his-3^K874^ cog+ x his-3^K1201^ cog*** |
| T10998 x T12708 | 760 | 402 | 945 | 58 | *Msh-2^+^/Δmsh-2* |
| T10998 x T12709 | 479 | 410 | 584 | 39 |  |
| T10998 x T12710 | 296 | 215 | 688 | 62 |  |
| Mean |  |  | 690 |  |  |
|  |  |  |  |  |  |
| T12298 x T12708 | 834 | 271 | 1539 | 108 | *Δmsh-2/Δmsh-2* |
| T12298 x T12709 | 766 | 376 | 1019 | 64 |  |
| T12298 x T12710 | 453 | 201 | 1127 | 96 |  |
| Mean |  |  | 1228 |  |  |
|  |  |  |  |  |  |
| T11089 x T11805 | 445 | 238 | 280 | 23 | ***his-3^K874^ cog x his-3^K1201^ cog^+^*** |
| T12705 x T11805 | 145 | 199 | 364 | 40 | *Msh-2^+^/Δmsh-2* |
| T12706 x T11805 | 144 | 203 | 355 | 39 |  |
| T12707 x T11805 | 108 | 96 | 563 | 79 |  |
| Mean |  |  | 390 |  |  |
|  |  |  |  |  |  |
| T12705 x T12299 | 196 | 265 | 370 | 35 | *Δmsh-2/Δmsh-2* |
| T12706 x T12299 | 187 | 238 | 393 | 38 |  |
| T12707 x T12299 | 206 | 199 | 518 | 51 |  |
| Mean |  |  | 427 |  |  |
|  |  |  |  |  |  |
